# Altering gait by way of stimulation of the plantar surface of the foot: the immediate effect of wearing textured insoles in older fallers

**DOI:** 10.1186/1757-1146-5-11

**Published:** 2012-04-30

**Authors:** Anna L Hatton, John Dixon, Keith Rome, Julia L Newton, Denis J Martin

**Affiliations:** 1 School of Health and Rehabilitation Sciences, The University of Queensland, Brisbane, QLD, Australia; 2 Centre for Rehabilitation Sciences, Health and Social Care Institute, Teesside University, Middlesbrough, UK; 3 Health & Rehabilitation Research Institute, AUT University, Auckland, New Zealand; 4 Institute for Ageing and Health, University of Newcastle, Newcastle-upon-Tyne, UK; 5 Health & Rehabilitation Research Institute, School of Podiatry, AUT University, Private Bag 92066, Auckland, New Zealand

**Keywords:** Textured insoles, Gait, Double-limb standing

## Abstract

**Background:**

Evidence suggests that textured insoles can alter gait and standing balance by way of enhanced plantar tactile stimulation. However, to date, this has not been explored in older people at risk of falling*.* This study investigated the immediate effect of wearing textured insoles on gait and double-limb standing balance in older fallers.

**Methods:**

Thirty older adults >65 years (21 women, mean [SD] age 79.0 [7.1]), with self-reported history of ≥2 falls in the previous year, conducted tests of level-ground walking over 10 m (GAITRite system), and double-limb standing with eyes open and eyes closed over 30 seconds (Kistler force platform) under two conditions: wearing textured insoles (intervention) and smooth (control) insoles in their usual footwear.

**Results:**

Wearing textured insoles caused significantly lower gait velocity (*P* = 0.02), step length (*P* = 0.04) and stride length (*P* = 0.03) compared with wearing smooth insoles. No significant differences were found in any of the balance parameters (*P* > 0.05).

**Conclusions:**

A textured insole worn by older adults with a history of falls significantly lowers gait velocity, step length and stride length, suggesting that this population may not have an immediate benefit from this type of intervention. The effects of prolonged wear remain to be investigated.

## Background

Providing enhanced sensory input to the plantar surface of the feet has recently been considered a potential mechanism through which footwear interventions may improve gait [1-8] and standing balance [9-12], by way of altering sensorimotor function.

Adding texture to the upper surface of shoe insoles (or floor surfaces) has been hypothesised to increase sensory afferent feedback via enhanced tactile stimulation of plantar cutaneous mechanoreceptors [9,10,13-16]. The literature on the effect of texture covers a range of populations, other than older people, reporting improved balance in healthy young adults [9,10,16]; no clear benefit to balance in healthy young adults [13] and middle-aged females [17]; and poorer balance in young adults with chronic ankle instability [15]. Two studies have been carried out on healthy older people, and their findings of beneficial effects on standing balance, when older people stood on a textured surface [14] and wore sandals with textured insoles [10], raise the possibility that textured footwear interventions could also affect gait in older adults with a history of falls.

The aim of this study was to evaluate the immediate effect of wearing textured insoles (compared with smooth insoles) on gait and standing balance in older adults with a history of falls.

## Methods

### Design

Within-subject experimental design with all participants tested under each of two conditions: (1) Intervention: wearing textured insoles in their usual footwear, and (2) Control: wearing smooth insoles in their usual footwear.

### Participants

30 participants (21 women) were included in the study. Inclusion criteria were community-dwelling older adults >65 years old with a self-reported history of ≥2 falls in the previous year. Participants were recruited through clinical staff working within local and regional National Health Service Falls and Elderly Care Services, UK. Exclusion criteria were self-reported neuromuscular disease, stroke, current history of peripheral neuropathy, or a Mini Mental State Examination (MMSE) score <27. All participants gave written, informed consent. Ethical approval was granted by the School of Health Research Governance and Ethics Committee at Teesside University, UK.

### Outcome measures

Gait measurements comprised velocity, cadence, step length, stride length, base of support, step time, cycle time, swing time, stance time, and single- and double-limb support times, and were obtained using GAITRite (CIR Systems, Inc., Havertown, PA 19083, USA). The GAITRite instrumentation has been reported to have high reliability in older adults [18] and high concurrent validity when compared with video-based motion analysis systems for spatial and temporal parameters of gait such as velocity, cadence, and stride length [19].

Double-limb standing balance measures were range and standard deviation of the centre of pressure (CoP) excursion in the mediolateral (ML range and ML SD) and anterior-posterior direction (AP range and AP SD), and CoP velocity. Double-limb standing balance data were extracted from a Kistler force platform (Model 9286AA, Kistler Instruments Ltd., Hampshire, UK) (sampling rate 1000 Hz).

### Materials

**Figure 1:**
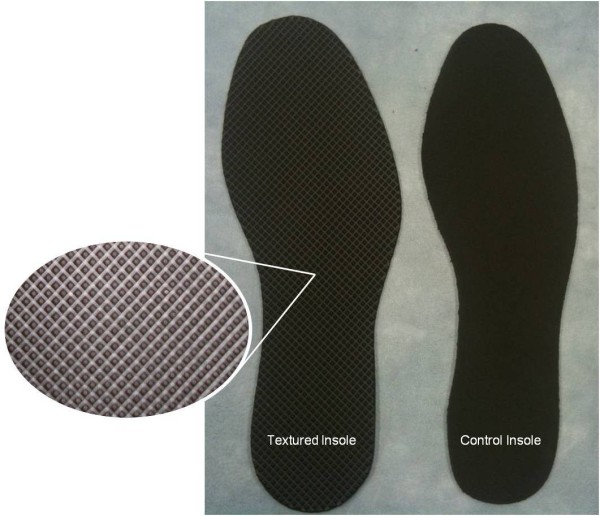
Textured and control insoles.

**Figure 2:**
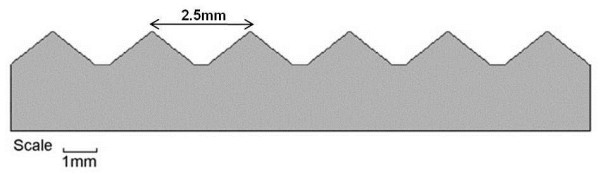
Cross-sectional frontal view of textured insole.

The textured insoles (Evalite Pyramid EVA, 3 mm thickness, shore value A50, black, OG1549) had small, pyramidal peaks with centre-to-centre distances of approximately 2.5 mm and were selected from a range of EVA soling materials (Algeos UK, Liverpool, UK) [13,14,17]. The control insoles (Medium Density EVA, 3 mm thickness, shore value A50, black, OG1304) had a completely flat surface (Figures 1,2). All insoles were cut to a range of men’s and women’s standard UK shoe sizes, at a local orthotic manufacturing workshop (Peacocks Medical Group, Newcastle-upon-Tyne, UK).

### Procedures

Testing was carried out in a university laboratory and all data collection was carried out by the same investigator (ALH). Neither the investigator nor the participants were blind to the condition being tested. Participants were asked to remove all hosiery for the tests. Participants completed the 12-Item World Health Organization Disability Assessment Schedule II (12-Item WHODAS II) [20] and scored average pain levels in the last three months, and pain at the time of testing, on separate 0–10 numerical rating scales for the upper limbs, back, feet/ankles and hips/knees [21].

Participants were not familiarised with the textured insoles prior to testing. It remains unknown whether a familiarisation period is required, how long this would take, and whether habituation to the textured stimuli would occur, in older fallers. Therefore, in the first instance, it was important to establish the immediate effects of wearing textured insoles on gait and balance, in this population, However, before data collection, each participant had a practice standing balance and walking trial wearing the control insoles, to ensure familiarity with the procedures. Then, wearing either the textured or smooth insoles, the individual participant’s standing balance was tested first followed immediately by gait analysis wearing the same insole. There was a two minute rest period to change insoles. The participant then wore the other insoles, and was tested again in standing balance followed by gait. Within the standing balance tests, all measures were taken with either eyes open or closed. The sequence in which the textures were worn and the eyes open/eyes closed sequence were determined by way of simple randomisation, in which the participant selected number cards from a sealed, opaque envelope. All tests were carried out during one session.

#### Double-limb standing balance

Balance measures were taken in four trials, one trial [22,23] for each of four conditions: textured insoles/eyes closed, textured insoles/eyes open, control insoles/eyes closed and control insoles/eyes open. To begin each trial, the participant stood up from a height-adjustable seat without trunk/back support or armrests. To standardise lower limb joint angles the seat height was standardised to knee height (the distance from the centre of the knee joint to the ground when standing in usual footwear) [24]. Once the participant was vertical, the investigator pressed a trigger which started recording from the Kistler force platform for 30 seconds. Between each trial, participants rested for 2 minutes to avoid fatigue and to allow re-calibration of the force platform.

#### Gait

Gait was measured in two trials, one trial [22,23] for each of the two conditions: textured insoles and control insoles. With the allocated insoles in place the participant stood up from a chair at the start line, and once standing began walking at their preferred, comfortable speed until told to stop, when both feet had passed over the finishing line. During walking tests, the investigator walked behind and to the left of participants to ensure their safety while remaining out of their direct view.

### Data extraction and analysis

Gait measurements were processed using GAITRite software. ML and AP CoP excursion variables (mm) were produced automatically by the force platform using the Bioware software. CoP velocity (mm.s^-1^) was calculated using previous methods [25], after low-pass filtering of the raw data at 10 Hz. Data from the last 20 seconds of testing were used for analysis. The first 10 seconds of data may exhibit unwanted variability due to stabilisation of participants [26] or the force platform [25], and were discarded [14,17,25].

### Statistical analysis

Data were analysed with SPSS version 17.0 (SPSS Inc, Chicago, IL 60606, USA). For each balance variable, and for gait velocity and cadence, a paired-samples t-test compared the two conditions. For the other gait measures, which provided figures for both left and right limbs, we used separate 2-way ANOVAS with texture and limb as within-subject factors. All tests were two-tailed with alpha at 0.05.

## Results

Thirty participants were recruited with a mean (SD): age 79.0 (7.1), range 66–92 years; height 163.0 (0.1) cm; weight 74.9 (14.0) kg; BMI 26.9 (4.0) kg/m^2^, MMSE 28.3 (4.4). The mean (SD) 12-Item WHODAS II disability score was 14.9 (9.3) from a possible range of 0–48 (using a simple summation of individual items), with a high score indicating high disability. Twenty-three of the 30 participants (77%) stated that they had at least moderate pain (≥4/10) in two or more joints in the last three months. All participants completed every trial.

### Gait

Table 1 shows the gait analysis results. Gait measurements were calculated using a sample of n = 26. The reduced sample size was due to corrupt data which had to be excluded from the analysis. No significant texture by limb interactions were observed, nor were there any significant limb main effects. Velocity was statistically significantly lower with the textured insoles compared to the control insoles (*P* = 0.02). Step length was statistically significantly shorter with the textured insoles compared to control (*P* = 0.04) independent of limb. Similarly, relative to control, stride length was statistically significantly shorter with the textured insoles (*P* = 0.03) independent of limb. No statistically significant differences were found for any other gait measurements (*P* > 0.05).

**Table 1:** Gait measurements for control and textured insoles during level-ground walking (N = 26)

**Gait measurement**	**Control**	**Texture**	***P*value**
	**Mean (SD)**	**Mean (SD)**	
Velocity (cm.s^−1^)	66.3 (27.0)	62.1 (26.6)	0.02*
Cadence (steps/min)	90.4 (17.5)	87.7 (17.7)	0.14
Step Time (Left Foot) (s)	0.7 (0.1)	0.7 (0.1)	insole 0.21
Step Time (Right Foot) (s)	0.7 (0.2)	0.7 (0.2)	limb 0.81
			interaction 0.72
Cycle Time (Left Foot) (s)	1.4 (0.3)	1.4 (0.3)	insole 0.21
Cycle Time (Right Foot) (s)	1.4 (0.3)	1.4 (0.3)	limb 0.62
			interaction 0.88
Step Length (Left Foot) (cm)	42.6 (11.9)	41.4 (12.4)	insole 0.04*
Step Length (Right Foot) (cm)	43.0 (12.1)	41.2 (11.9)	limb 1.00
			interaction 0.66
Stride Length (Left Foot) (cm)	85.5 (23.9)	82.6 (23.8)	insole 0.03*
Stride Length (Right Foot) (cm)	86.1 (23.9)	83.2 (24.8)	limb 0.09
			interaction 0.94
Heel to Heel Base (Left Foot) (cm)	12.3 (4.5)	10.0 (8.6)	insole 0.24
Heel to Heel Base (Right Foot) (cm)	12.1 (4.5)	10.2 (8.2)	limb 0.87
			interaction 0.18
Single Support Time (Left Foot) (%GC)	31.5 (5.2)	30.7 (5.3)	insole 0.14
Single Support Time (Right Foot) (%GC)	31.5 (5.4)	31.0 (5.3)	limb 0.89
			interaction 0.68
Double Support Time (Left Foot) (%GC)	37.9 (9.0)	38.8 (9.4)	insole 0.21
Double Support Time (Right Foot) (%GC)	37.8 (8.9)	38.7 (9.7)	limb 0.52
			interaction 0.90
Swing Time (Left Foot) (%GC)	31.5 (5.4)	31.2 (5.5)	insole 0.15
Swing Time (Right Foot) (%GC)	31.5 (5.1)	30.6 (5.1)	limb 0.70
			interaction 0.42
Stance Time (Left Foot) (%GC)	68.5 (5.3)	68.8 (5.5)	insole 0.14
Stance Time (Right Foot) (%GC)	68.6 (5.1)	69.5 (5.1)	limb 0.69
			interaction 0.41

The table shows *P* values of paired t-tests for velocity and cadence. For the other gait variables it shows *P* values of 2-way ANOVA main effects of insole and limb, and insole x limb interaction. * *P* < 0.05.

### Double-limb standing balance

Table 2 shows the results with eyes open and closed during the latter 20 seconds of double-limb standing. There were no statistically significant effects of the textured insoles on AP or ML range or SD or CoP velocity, with eyes open or closed (*P* > 0.05).

**Table 2:** Balance measurements for control and textured insoles during double-limb standing (N = 30)

Balance measurement	**Eyes open**			**Eyes closed**		
	**Control**	**Texture**	***P*value**	**Control**	**Texture**	***P*value**
	**Mean (SD)**	**Mean (SD)**		**Mean (SD)**	**Mean (SD)**	
AP SD (mm)	5.9 (2.4)	6.3 (2.6)	0.444	6.9 (3.0)	6.6 (2.5)	0.403
AP range (mm)	31.7 (10.5)	35.8 (14.5)	0.178	37.4 (17.8)	36.5 (14.1)	0.638
ML SD (mm)	4.5 (2.1)	5.0 (2.9)	0.297	5.0 (4.5)	4.2 (3.0)	0.167
ML range (mm)	25.8 (12.7)	27.4 (13.7)	0.548	26.8 (17.7)	24.8 (17.6)	0.398
CoP velocity (mm.s^−1^)	19.7 (11.0)	18.6 (8.4)	0.591	24.8 (15.3)	27.8 (20.3)	0.245

## Discussion

This study aimed to investigate the immediate effect of wearing textured insoles on gait and double-limb standing balance in older people with a history of falling. Gait velocity, step length and stride length were significantly reduced when wearing the textured insoles. We did not find any significant differences between the control and textured insoles in any of the standing balance parameters.

To the authors’ knowledge, one previously published study explored the effect of a footwear intervention on gait in older fallers [1], showing significant improvements in gait timing variability when walking in vibrating shoe insoles. The difference between the previous work [1] and the current study may be related to the characteristics of the sensory stimuli. Galica et al. [1] used sub-threshold vibratory sensors in the insole, whilst the current study used textured insoles. In the current study, the plantar surface of the feet remained in contact with the indentations of the textured insole: this may have stimulated slow-adapting mechanoreceptors, which are reported to respond to maintained and prolonged skin indentation [27]. In comparison, vibrotactile footwear interventions permit manipulation of the frequency, intensity, phase and duration of stimuli [28], and may consequently affect fast-adapting mechanoreceptors which show burst responses to stimuli, only at the point of application or removal from the foot [27]. Vibratory stimuli may also affect intrinsic foot proprioceptors, in addition to cutaneous mechanoreceptors [29]. It is unlikely that textured insoles work on this same principle as they provide neither mechanical nor electrical stimuli.

Furthermore, the primary outcome variables were also different between our study and Galica et al. [1], who measured gait variability. In the current study we did not calculate gait variability, as there is lack of consensus as to which measures of variability are most useful when quantifying changes in gait performance. The mean baseline gait speed of Galica et al. [1] was over 100 cm.s^-1^ compared to 66.3 cm.s^-1^ in the current study. However, baseline gait speed of older fallers in this study was similar to that reported for frail older people in previous work [30-32]. Therefore, we could speculate that older people with substantially impaired gait performance at baseline, may not benefit from this type of intervention, but for those with less impairment, footwear interventions may be more useful.

There was no significant difference of the textured insole on standing balance, unlike in our previous study with healthy older people, which showed statistically significant effects with the same texture, when used as a floor surface [14]. It is unclear whether this is due to the delivery of the textured intervention: as a floor surface versus an insole. There is much debate relating to the effect of footwear features on postural stability. Menant et al. [33] reported that shoe characteristics, including an elevated heel and high-heel collar, could alter balance performance in older people. In comparison, Horgan et al. [34] concluded that footwear features were not associated with changes in balance scores in older women. In the current study, it is possible that incorporating a textured insole into footwear may have brought about a dampening effect of the sensory stimulus, contributing to the non-significant findings. However, the fact that participants were measured using their own shoes, and that benefits were independent of shoe types, makes the results more generalizable and relevant to daily life. Lack of agreement between the current study findings and our previous work [14] may also be due to differential effects between the two populations; or due to relatively greater variance in the current data masking similarly small effect sizes.

It may be that the older fallers in the current study had difficulty processing the extra sensory information, delivered through a new medium, the textured insoles, which could have constrained the sensorimotor system [15]. It may be that the unfamiliar sensory stimulus on the plantar surface of the foot may have caused older people to walk more cautiously (with shorter steps and at a slower velocity), rather than produce an actual decline in balance ability.

The study has a number of limitations. Due to the lengthy test procedures, we did not want to risk fatigue in this first study with older fallers and, therefore, used one repetition rather than multiple trials for gait and standing balance tests. Whilst this may have resulted in relatively large measurement error in the data, it appears that the magnitude of the textured effect on gait was larger than the measurement error, suggesting this is a true finding. Our findings are specifically about the effects when the insoles were worn for the first time and should not be extrapolated outwith that context, as we did not investigate the effects of prolonged exposure to the insoles. The finding that textured insoles had an immediate effect on gait variables in older fallers supports the need for further investigation into their long-term effects.

## Conclusions

These results show that stimulating the plantar surface of the foot by way of wearing this type of textured insole leads to a significant reduction in gait velocity, step length and stride length. The effects of textured insoles in older adults with a history of falls may not be beneficial immediately. The effects of prolonged wear remain to be investigated.

## Competing interests

The authors declare that they have no competing interests.

## Authors’ contributions

ALH, JD, KR, JLN and DJM conceived and designed the study. ALH collected and inputted the data. ALH, JD and DJM conducted the statistical analysis. ALH, JD, KR, DJM and JLN compiled the data and drafted the manuscript. All authors read and approved the final manuscript.
